# Spatial Metabolomics Reveals the Effects of Dietary Capsaicin Intervention on Interscapular Adipose Tissue Metabolome in Mice

**DOI:** 10.3390/foods13233943

**Published:** 2024-12-06

**Authors:** Haoqing Yang, Peiying Zheng, Jiamiao Hu, Zhongjing Lin, Natthida Sriboonvorakul, Shaoling Lin

**Affiliations:** 1 College of Food Science, Fujian Agriculture and Forestry University, Fuzhou 350002, China; haoqing-yang@fafu.edu.cn (H.Y.); zhengpy19990520@163.com (P.Z.); jiamiaohu@fafu.edu.cn (J.H.); zhongjing.lin@fafu.edu.cn (Z.L.); 2 Engineering Research Centre of Fujian-Taiwan Special Marine Food Processing and Nutrition, Ministry of Education, Fuzhou 350002, China; 3 Department of Clinical Tropical Medicine, Faculty of Tropical Medicine, Mahidol University, Bangkok 10400, Thailand

**Keywords:** spatial metabolomics, capsaicin, lipid profile, adipose tissue

## Abstract

Capsaicin is a polyphenol with a well-known anti-obesity potential, which could activate brown adipose tissue and promote the browning of white adipose tissue. Indeed, conventional proteomics have been used to investigate the browning effects of capsaicin on adipose tissue. However, the existence of a layer of white adipose tissue above the interscapular brown adipose tissue poses a great challenge to obtain intact interscapular brown adipose tissue without including adjacent white adipose tissue. Therefore, the traditional method normally focuses on changes occurring in the bottom layer of interscapular brown adipose tissue. Spatial metabolomics is an omics method that enables the analysis of metabolite distributions in tissue sections. Therefore, in the current study, spatial metabolomics was utilized to investigate the effects of dietary capsaicin intervention on interscapular brown adipose tissue and adjacent white adipose tissue. The results indicated several noteworthy findings that capsaicin treatment may induce similar metabolite alterations across various regions of brown adipose tissue irrespective of their proximity to WAT, while it also markedly influences the metabolites in the adjacent white adipose tissue. A KEGG pathway analysis further revealed these changes were associated with key characteristics of beige energy metabolism pathways, such as thermogenesis, glycerol phospholipid metabolism, and pentose phosphate pathway. Taken together, this study may supplement useful details to understand the mechanisms of capsaicin enhancing BAT activity and promoting WAT browning.

## 1. Introduction

Obesity has become an epidemic disease that greatly elevates the risk of developing serious health conditions such as cardiovascular disease [1], high blood pressure [2], diabetes [3], and atherosclerosis [4]. Indeed, obesity is often linked with adipose tissue expansion, which mainly results from the enlargement of existing adipocytes (adipocyte hypertrophy) or the formation of new adipocytes (adipocyte hyperplasia) [5]. Notably, mammalian adipose tissue could be divided into white adipose tissue (WAT) and brown adipose tissue (BAT) [6]. WAT mainly stores excess energy in the form of triglycerides [7]. At the same time, BAT has the remarkable capability to generate large amounts of heat by dissipating excess energy via non-shivering thermogenesis [8], a process primarily mediated by uncoupling protein 1 (UCP-1) [9]. Since the discovery of BAT in adult humans, the activation of BAT and the induction of WAT browning have been recognized as promising therapeutic strategies for treating obesity [10].

Currently, several natural bioactive compounds have been documented as being capable of activating BAT or inducing the browning of WAT [11]. For instance, dietary supplementation with curcumin was found to elicit subcutaneous white adipose tissue (sWAT) browning, thus mitigating body fat gain and metabolic disturbances in rats [12]. Selenium-enriched green tea polysaccharides were also reported to stimulate inguinal white adipose tissue (iWAT) browning and enhance BAT thermogenesis [13]. Similarly, capsaicin supplementation was also found to directly stimulate BAT and induce the browning of WAT [14]. Notably, among these bioactive compounds, the underlying mechanism by which capsaicin activates BAT and induces WAT browning has been well elucidated, which mainly involves the activation of transient receptor potential vanilloid 1 (TRPV1), a non-selective cation channel. When capsaicin binds to TRPV1, it leads to an increase in intracellular calcium (Ca^2+^) levels. This rise in Ca^2+^ further triggers a series of cellular events, including the phosphorylation of Ca^2+^/calmodulin-activated protein kinase II (CaMKII) and AMP-activated protein kinase (AMPK). These signaling pathways, in turn, enhance the expression and activity of sirtuin-1, a protein that plays a crucial role in energy metabolism. Through this mechanism, capsaicin encourages the transformation of WAT into a more metabolically active, brown-like state [15].

As the lipid storage depot, different types of adipose tissues exhibit distinct lipid compositions. For instance, Hoene et al. demonstrated that BAT in mice contains a higher abundance of phospholipids (phosphatidylethanolamines (PEs) and phosphatidylcholines (PCs)) and free fatty acids compared to subcutaneous and perigonadal WAT, while WAT displays a greater presence of diacylglycerols (DAGs) and triacylglycerols (TAGs) than BAT [16]. Another lipidomic investigation comparing the primary white, beige, and brown adipocytes revealed that thermogenic adipocytes contain higher contents of phosphatidylethanolamine (PE) and phosphatidylcholine (PC) fractions as well as cardiolipin (CL), in comparison to white adipocytes [17]. Indeed, the differences in the lipid composition may be also intricately associated with the functional and morphological distinctions between WAT and brown/beige adipose tissues. For example, abundant free fatty acids in BAT enable their rapid utilization for FA oxidation [18], whereas WAT possesses more TAGs due to its role in energy storage [19].

Notably, the lipid compositions in adipose tissues could be influenced or even remodeled by various physiological conditions, environmental stimuli, or dietary intervention [20]. Consequently, the application of metabolomics has emerged as a powerful approach for elucidating the regulatory roles of bioactivities on adipose tissues. For instance, metabolomics studies have provided insights into cellular transition occurring during the browning of WAT upon zafirlukast stimulation [21]. Hou et al. also utilized liquid-chromatography-mass spectrometry (LC-MS) to investigate how *Tupaia belangeri* responds to low-temperature stress by regulating the concentrations of various metabolic pathway intermediates in WAT and BAT [22]. Additionally, mass spectrometry/mass spectrometry ALL (MS/MSALL) shotgun lipidomics has also been successfully employed to investigate the impact of exercise training on the lipidome of sWAT and BAT [23].

Nonetheless, there still exist certain methodological challenges in thoroughly assessing the impact of dietary intervention on the metabolic profiles in intact interscapular brown adipose tissue. For instance, the presence of a layer of white adipose tissue above the interscapular brown adipose tissue in mice presents a significant impediment in obtaining intact interscapular brown adipose tissue while avoiding the inclusion of adjacent white adipose tissue. Spatial metabolomics represents an innovative approach that extends the metabolomic analysis to a three-dimensional level, which enables the investigation of the spatial distribution of small molecules in tissue sections and uncovers intercellular heterogeneity. Up-to-date, spatial metabolomics has been successfully applied to various tissues such as the liver [24], kidney [25], skeletal muscle [26], lung [27], and plant bodies [28]. However, to the best of our knowledge, there have been no reports yet that have employed spatial metabolomics techniques in the context of iBAT. Therefore, in the current study, spatial metabolomics was adopted to analyze the changes in the metabolome of iBAT and its adjacent WAT upon capsaicin administration.

## 2. Materials and Methods

### 2.1. Animal Studies

Four-week-old C57BL/6J mice (male) were obtained from Wushi Experimental Animals Co., Ltd. (Fuzhou, Fujian, China), and maintained under controlled environmental conditions (23 °C ± 1 °C, relative humidity 55% ± 5%, 12 h light/dark cycle) with free access to a normal diet (Supplementary Table S1) and water. Following a one-week adjustment, the mice were divided into two groups: the Con group (control, which received a normal diet + saline) and the Cap group (experimental, which was administered a normal diet supplemented with capsaicin). Capsaicin (Sigma-Aldrich) was dissolved in sterile water to prepare a stock solution (5 mg/mL, stored at −80 °C); the working fluid concentration was 0.1 mg/mL. The mice in the Cap group were orally administered capsaicin at 2 mg/kg body weight for 4 weeks. Body weight and food consumption were assessed every three days. Thermal imaging was captured to evaluate the thermogenic capacity of mice. On the 28th day, all mice underwent a 12 h fasting period before the oral glucose tolerance test (OGTT) was conducted. The mice were euthanized by placing them in a chamber filled with gradually increasing concentrations of carbon dioxide (CO_2_) and then sacrificed by cervical dislocation. Immediately thereafter, the interscapular adipose tissue (as well as the adjacent white adipose pad) was removed and immersed in the embedding agent (Cryo-Gel). All animal experiments reported in the current study were approved by the Animal Management and Use Committee of Fujian Agriculture and Forestry University [No. PZCASFAFU22037] and adhered to the National Institutes of Health Guide for the Care and Use of Laboratory Animals.

### 2.2. Serum Lipid Levels

Blood samples were collected from the eyeballs immediately followed by the sacrifice of the mice, and then centrifuged at 3000 rpm to isolate the serum. The concentrations of triglyceride (TG), total cholesterol (TC), high-density lipoprotein cholesterol (HDL-C), and low-density lipoprotein cholesterol (LDL-C) were quantified by an automatic biochemical analyzer (Hitachi High-Technologies Corporation, Tokyo, Japan).

### 2.3. Mass Spectrometry Imaging Data Acquisition

#### 2.3.1. Slice Pre-Processing

Tissue samples were removed from the −80 °C ultra-low temperature freezer and allowed to thaw in the −20 °C freezer overnight. Afterward, the samples were sectioned into 30 μm slices and attached to Superfrost Plus (Thermo Fisher Scientific, Waltham, MA, USA) positively charged anti-dehiscence slides. The sequential sections were obtained, with one for hematoxylin-eosin staining (HE staining) and another for subsequent imaging analysis.

#### 2.3.2. Configuration of Solutions

Acetonitrile/water = 80:20 (*v*/*v*, containing 0.1% formic acid) was used as the electrospray solvent in positive ion mode, while the electrospray solvent in negative ion scanning mode was acetonitrile/water = 80:20 (*v*/*v*). The solvent flow rate was 5 μL/min.

#### 2.3.3. Data Acquisition

The scanning parameters were set as follows: Vx was 0.2 mm/s, Dy was 0.1 mm, Dt was 7 s, and the scanning region covered a length of 10 mm along both the X-axis and Y-axis. The mass spectrometry imaging data were acquired using a progressive scanning method, which ensured the accuracy and reliability of the mass spectrometry imaging results. The Xcalibur system was used for data acquisition and processing. The data acquisition sequence was determined by the sample size, step spacing, and scanning speed. Before multivariate analysis, the data were log-transformed and normalized by using Pareto scaling. An unsupervised principal component analysis (PCA) was utilized to analyze the general distribution amongst samples and the stability of the entire analytical process, followed by a supervised orthogonal partial least squares analysis (OPLS-DA) to distinguish the overall distinctions in metabolic profiles amongst groups. To prevent model overfitting, 7-fold cross-validation and 200-response permutation testing (RPT) were used to examine the quality of the model.

The VIP (variable important in projection) value in OPLS-DA analysis was used to measure the intensity and explanatory power of the expression pattern of each metabolite on the categorical discrimination of each group of samples. The *t*-test was further used to verify whether the metabolites were significant between groups. Metabolites with VIP values > 1 for the first principal component and a *p*-value < 0.05 for the *t*-test were screened through OPLS-DA analysis.

Metabolite Annotation was performed using the SmetDB database (Small Molecule Enrichment Tool Database) with pySM (Python-based small molecule pathway analysis). The metabolites were converted to KEGG-compatible formats and imported into KEGG Pathway Tools for KEGG pathway analysis.

### 2.4. Statistical Analysis

The data were statistically analyzed using Graphpad Prism 8.0.1 (Graphpad Software Inc., La Jolla, CA, USA) and are expressed as mean ± standard deviation (SD). Significant differences were determined using Student’s *t*-test or an ANOVA with Tukey’s post hoc test. Differences were considered significant at *p* < 0.05.

## 3. Results

### 3.1. Effect of Capsaicin Intervention on Physiological and Metabolic Characteristics of Normal Diet-Fed Mice

**Figure 1:**
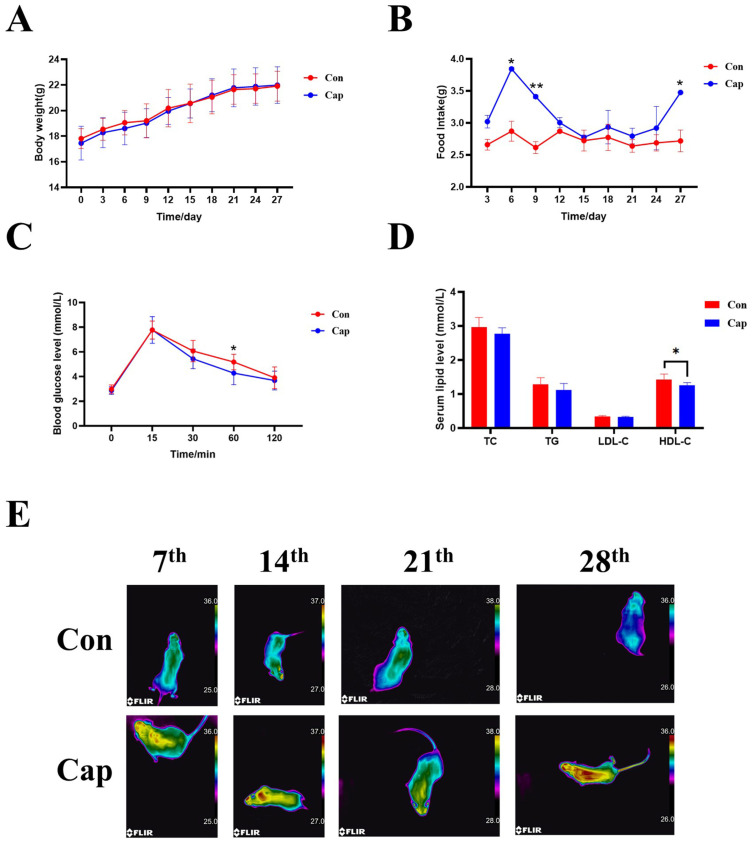
The influence of capsaicin treatment on the body weight (**A**), food intake (**B**), oral glucose tolerance test (**C**), and serum lipid level of mice (**D**), and the representative thermal image (**E**) of mice (*n* = 8). Con, control group; Cap, capsaicin-treated group. Data were expressed as the mean ± SD. * *p* < 0.05 and ** *p* <0.01 vs. Con group.

As depicted in Figure 1A, there was no significant difference in body weight gain between the control and experimental groups throughout the study. However, there existed a statistically significant increase (*p* < 0.05) in food consumption upon long-term capsaicin treatment (Figure 1B). This observation implies that long-term capsaicin supplementation may augment the energy expenditure of mice to counteract the impact of increased food intake on body weight gain.

Further, the influences of capsaicin supplementation on glucose tolerance and serum lipid levels were also determined. As illustrated in Figure 1C, mice in both groups exhibited an upward trend in blood glucose levels within the initial 15 min following the oral administration of force-fed glucose. Then, the blood glucose levels started to decline with capsaicin-treated mice outpacing the mice in the control group (reaching statistical significance at 60 min post-oral glucose administration). Finally, the blood glucose levels returned to pre-meal levels after two hours. This observed pattern implies the beneficial impact of capsaicin on glycemic homeostasis.

Capsaicin is also a well-recognized anti-obesity agent for reducing blood lipid levels in obese patients. Here, as shown in Figure 1D, in normal diet fed mice, no significant disparities in total cholesterol (TC), triglycerides (TG), or low-density lipoprotein cholesterol (LDL-C) levels were observed between the two groups, with significant, albeit slightly lower HDL-C levels being found in capsaicin-treated mice (*p* < 0.05).

To confirm the enhanced energy metabolism of capsaicin-treated mice, thermal images using an infrared thermography camera were taken to assess the body surface temperature among the mice in the two groups. The results showed that the mice in the experimental group exhibited a higher body temperature at the interscapular region than those in the control group, implying that the capsaicin-treated mice may have shown enhanced metabolic rates in interscapular BAT (Figure 1E).

### 3.2. Spatial Metabolomics Revealed the Effects of Capsaicin Treatment on the Metabolite Profile in Interscapular BAT and Adjacent WAT

#### 3.2.1. Definition of Tissue Anatomical Regions

**Figure 2:**
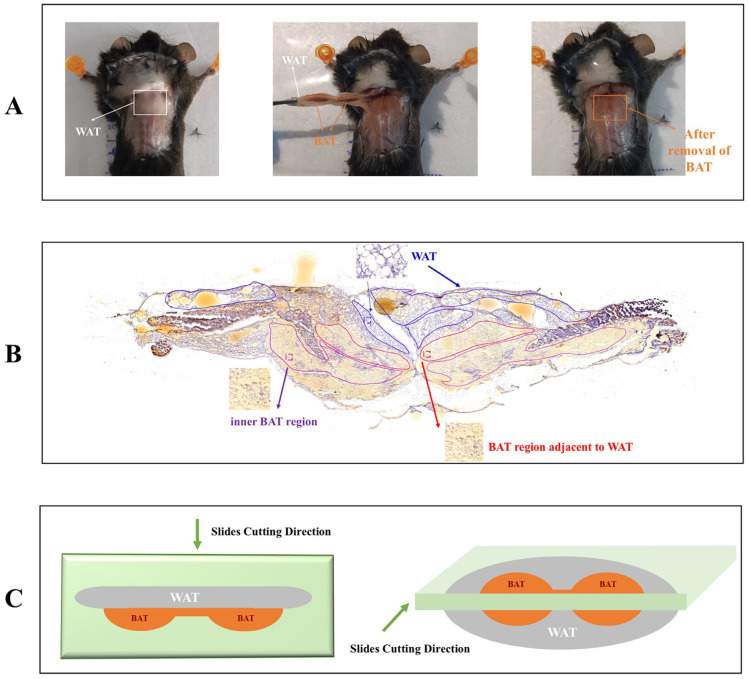
Anatomy schematic and defined anatomical tissue regions. (**A**) Representative images of removal of interscapular BAT and adjacent WAT. Left: top view of interscapular BAT regions; middle: isolation of interscapular BAT and adjacent WAT; right: images after removal of interscapular BAT and adjacent WAT; (**B**) representative images of partitioning the WAT region, BAT region adjacent to WAT, and inner BAT region (bottom layer) according to the HE staining result. (**C**) Schematic diagram of slice cutting directions for spatial metabolomics (*n* = 3).

Consistent with the anatomical observation (Figure 2A), the HE staining results also confirmed the presence of adipocytes with large lipid droplets (a hallmark feature of WAT) above the adipocytes containing small lipid droplets (a characteristic of BAT). Based on the HE results, the W region (WAT region, blue areas), B_inner region (inner BAT region, purple areas), and B_adj region (BAT region adjacent to WAT, red areas) were labeled for tissue slices in both the control and experimental groups (N = 3, respectively).

#### 3.2.2. Spatial Metabolomics Revealed the Metabolite Profile Differences Between Interscapular BAT and Adjacent WAT

**Figure 3:**
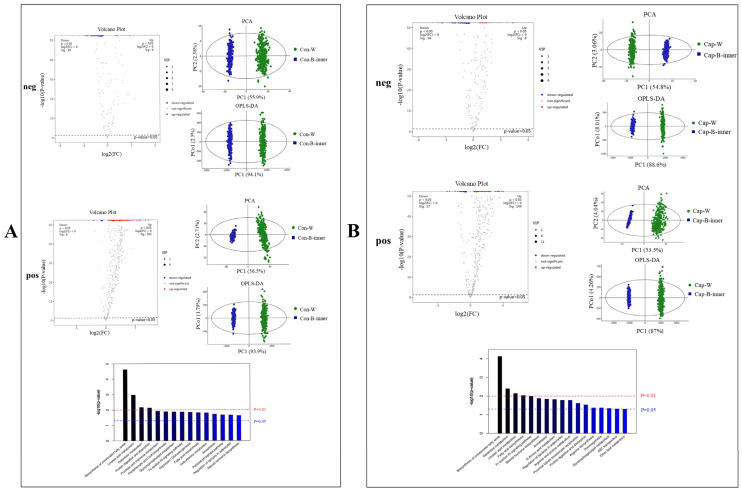
Volcano Plot, PCA, OPLS-DA, and KEGG pathway analysis of differential metabolites between WAT region and inner BAT region. (**A**) Con-W vs. Con-B-inner; (**B**) Cap-W vs. Cap-B-inner. “neg” refers to analyses obtained in negative ion mode and “pos” refers to analyses obtained in positive ion mode. The red line indicates *p* < 0.01, the blue line indicates *p* < 0.05 (*n* = 3).

We first compared the metabolite profile differences between the WAT region and inner BAT (B_inner) region of mice in the control group. The PCA and OPLS-DA analyses showed there existed an obvious separation between these two regions in both the control group and capsaicin-treated group (Figure 3). In mice without capsaicin treatment, after a comparison between the WAT region and inner BAT (Con_W vs. Con_B_inner), 464 significantly upregulated differential metabolites (8 in the Neg ion mode, and 456 in the Pos ion mode) and 147 significantly downregulated differential metabolites (140 the Neg ion mode and 7 in the Pos ion mode) were identified as shown in the volcano plot. Particularly, a range of lipid metabolites and lipid-related metabolites such as Oleamide, triglyceride (TG), Monoglyceride (MG), Phosphatidylserine (PS), Phosphatidic Acid (PA), nutriacholic acid, 3b-Hydroxy-5-cholenoic acid, as well as Histamine, Nicotine, and Dimethylethanolamine were found to be highly abundant in the cells with large lipid droplets (W region). In contrast, fatty acid esters of hydroxy fatty acids (FAHFAs), Lysophosphatidylethanolamine (LPE), 3-Oxo-4,6-choladienoic acid, Spermine, etc. were highly abundant in cells in the B_inner region (Supplementary Table S2). The KEGG analyses of identified differential metabolites between the W region and B_inner region further revealed that a number of pathways were significantly enriched, including the biosynthesis of unsaturated fatty acids, linoleic acid metabolism, galactose metabolism, protein digestion and absorption, fructose and mannose metabolism, pantothenate and CoA biosynthesis, glycerophospholipid metabolism, Fc epsilon RI signaling pathway, glycolysis/gluconeogenesis, fatty acid biosynthesis, beta-alanine metabolism, amoebiasis, pentose phosphate pathway, the regulation of lipolysis in adipocytes, and steroid hormone biosynthesis. The aforementioned differential metabolic pathways were closely related to energy metabolism, lipid synthesis, and catabolism.

Similarly, the results also revealed considerable differences between the WAT region and inner BAT region of mice with capsaicin treatment (Cap_W vs. Cap_B_inner). The results from the volcano map illustrated that the differences in metabolites between “Cap_W vs. Cap_B_inner” included 16 significantly up-regulated differential metabolites, 146 significantly down-regulated differential metabolites in the neg ion mode, and 297 significantly up-regulated differential metabolites and 21 significantly down-regulated differential metabolites in the pos ion mode (Supplementary Table S3). The KEGG results also showed that, similar to the comparison between Con_W vs. Con_B_inner in mice without capsaicin treatment, a number of pathways related to energy metabolism, fat synthesis, and catabolism were enriched. Particularly, it is noteworthy that among these enriched pathways, the “thermogenesis” pathway was identified, suggesting there exist different thermogenic activities between iBAT and the adjacent WAT upon capsaicin treatment.

In summary, based on the above findings, it is evident that WAT and BAT in both the control group and experimental group showed distinct metabolite features, indicating the partitioning of the regions according to HE staining was reasonable.

#### 3.2.3. Spatial Metabolomics Revealed the Effects of Capsaicin Treatment on the Metabolite Profile in Different Regions of Interscapular BAT

**Figure 4:**
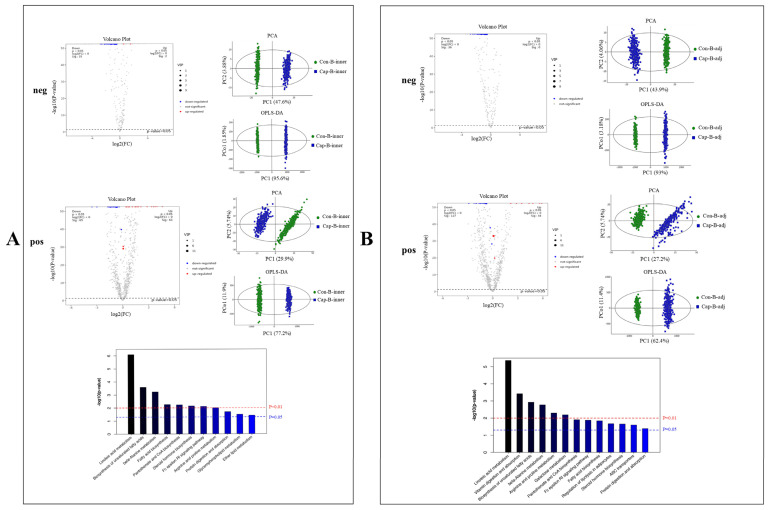
Volcano Plot, PCA, OPLS-DA, and KEGG pathway analysis of differential metabolites in BAT regions upon capsaicin treatment. (**A**) Con-B_inner vs. Cap-B_inner; (**B**) Con-B_adj vs. Cap-B_adj. “neg” refers to analyses obtained in negative ion mode and “pos” refers to analyses obtained in positive ion mode. The red line indicates *p* < 0.01, the blue line indicates *p* < 0.05 (*n* = 3).

Next, the metabolite profile changes in the B_inner region upon capsaicin treatment were evaluated (Figure 4A). As expected, a notable distinction could be observed between specimens of mice in the control group and the experimental group. In summary, 6 significantly up-regulated and 155 significantly down-regulated metabolites were obtained in the neg ion mode, with 123 significantly up-regulated and 134 significantly down-regulated metabolites in the pos ion mode, including a range of MG, DG, TG, FA, FAHFAs, etc. (Supplementary Table S4). The KEGG analysis also revealed that metabolic pathways including linoleic acid metabolism, the biosynthesis of unsaturated fatty acids, fatty acid biosynthesis, vitamin digestion and absorption, fructose and mannose metabolism, GnRH signaling pathway, leishmaniasis, Fc gamma R-mediated phagocytosis, long-term depression, and necroptosis were enriched.

**Figure 5:**
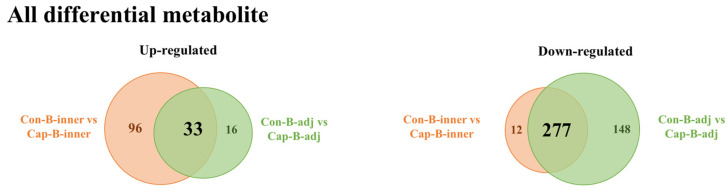
Venn diagram representing the overlap between the identified differential metabolite profiles of the B_inner regions and B_adj regions upon capsaicin treatment. (**Left**): up-regulated metabolites upon capsaicin treatment; (**right**): down-regulated metabolites upon capsaicin treatment.

Interestingly, more differential metabolites were observed from the B_adj region when comparing the two groups (Figure 4B). In total, 155 metabolites were significantly down-regulated in the neg ion mode and 49 were significantly up-regulated with 270 being significantly down-regulated in the pos ion mode (Supplementary Table S5). Notably, among these differential metabolites identified, nearly three-quarters (33 of 49 up-regulated metabolites and 277 of 425 down-regulated metabolites) exhibited overlap in altered metabolites when comparing the B_inner region (Figure 5). This high degree of overlap indicated that capsaicin intervention induced high similarities of metabolite alterations across different regions of BAT, and only slight differences persisted between these two areas.

#### 3.2.4. Spatial Metabolomics Revealed the Effects of Capsaicin Treatment on the Metabolite Profile in Adjacent WAT

**Figure 6:**
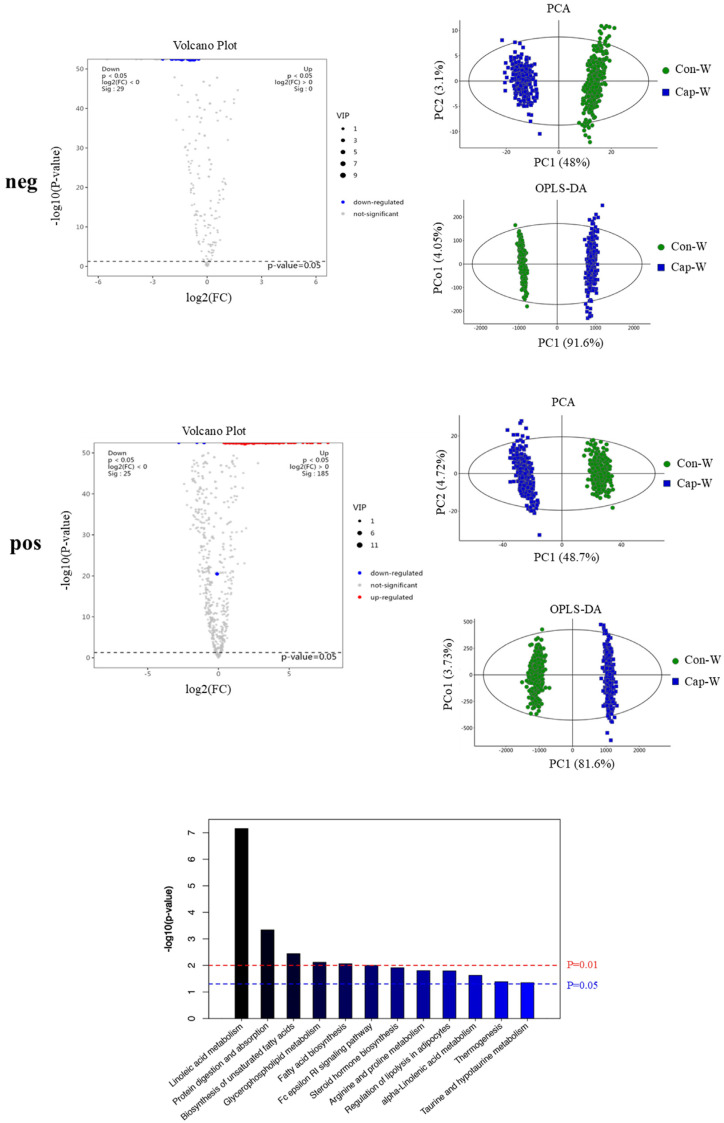
Volcano plot, PCA, OPLS-DA, and KEGG pathway analyses of differential metabolites between WAT regions upon capsaicin treatment. “neg” refers to analyses obtained in negative ion mode and “pos” refers to analyses obtained in positive ion mode. The red line indicates *p* < 0.01, the blue line indicates *p* < 0.05 (*n* = 3).

Meanwhile, the metabolite profile changes in the W region upon capsaicin treatment were also evaluated (Figure 6). Consistent with its browning-inducing activity, capsaicin intervention was capable of eliciting obvious changes in the metabolic fingerprint in the W region containing adipocytes with large lipid droplets. As shown in the volcano plot, 132 significantly down-regulated metabolites were identified in the neg ion mode, as well as 447 significantly up-regulated and 39 significantly down-regulated metabolites in the pos ion mode (Supplementary Table S6). Notably, these differential metabolites included a number of lipids that are closely linked to energy metabolism pathways, such as TG, DG, FA, and PC. These lipids play a crucial role in the process of the browning of WAT induced by capsaicin. KEGG results also showed that a number of metabolic pathways were enriched including linoleic acid metabolism, protein digestion and absorption, the biosynthesis of unsaturated fatty acids, glycerophospholipid metabolism, fatty acid biosynthesis, Fc epsilon RI signaling pathway, steroid hormone biosynthesis, arginine and proline metabolism, the regulation of lipolysis in adipocytes, alpha-linolenic acid metabolism, thermogenesis, and taurine and hypotaurine metabolism. Particularly, thermogenesis, a characteristic of brown adipose tissue, was identified, suggesting that capsaicin intervention may contribute to the browning of WAT by boosting the thermogenic and lipolytic capabilities.

## 4. Discussion

In this study, the effects of dietary capsaicin intervention on interscapular BAT were re-evaluated, with a particular focus on employing spatial metabolomics techniques. Although no significant difference in body weight between the two groups was observed, it was found that the food intake of mice with capsaicin treatment was higher than that in the control group. Indeed, capsaicin, as an adrenergic receptor agonist, has been confirmed to enhance energy consumption [29]. Thus, this might explain why increased food intake did not result in significant changes in body weight. Notably, in a previous study, Christy L. White et al. showed that capsaicin significantly reduced the food intake of male mice fed a high-fat diet or low-fat diet [30], while in another study, M Ghorbani reported that capsaicin did not alter the food intake of obese mice [31], suggesting that the influence of capsaicin treatment on food intake may be greatly influenced by a multitude of factors, such as the animal model utilized, the type of feeding provided, the environmental conditions, etc. In addition, infrared thermography also supports this hypothesis since capsaicin treatment leads to higher body surface temperatures.

It is generally believed that capsaicin is a good anti-obesity agent; therefore, the glucose tolerance and blood lipid levels of mice was also tested. The obtained results also demonstrated the beneficial influences of capsaicin treatment in mice evidenced by a faster decline in postprandial blood glucose levels. Although capsaicin has been well documented to reduce serum and liver lipid levels [32] and reduce weight [33] in obese mice, surprisingly, the effect of capsaicin on the blood lipid profile in our study was subtle, which may be attributed to the fact that the mice were fed with a normal diet.

Next, based on HE staining, the interscapular BAT and its adjacent WAT were divided into three types of regions: the W region (WAT region); B_inner region (inner BAT region), and B_adj region (BAT region adjacent to WAT). Notably, the distinct differences in metabolites identified between the W regions and B_inner regions corroborated the anatomical observation of a layer of WAT overlaying the interscapular BAT. This finding may also support our hypothesis that including the adjacent WAT in traditional metabolomics analyses may result in inaccuracies in the outcomes. In addition, our results also showed that alterations in metabolites exhibited highly similar patterns when analyzing both the B_inner region and B_adj region following capsaicin treatment. This finding indicated that interscapular BAT, as an intact organ, consistently responds to capsaicin treatment, irrespective of its proximity to WAT.

Meanwhile, our KEGG analysis also revealed that the thermogenesis pathway was enriched when comparing the different metabolites in the W region (WAT) in mice with or without capsaicin treatment. Similarly, several studies by P. Baskaran et al. [15] and R. K. Baboota et al. [34] also reported an up-regulation in thermogenic genes, including UCP1, CIDEA, PPARα, PGC1α, and SIRT1, in white adipose tissue (WAT) following capsaicin treatment [35]. This finding robustly corroborates the widely recognized phenomenon of ‘browning’ induced by capsaicin. Indeed, a number of metabolites in WAT altered by capsaicin were found to be lipid substances related to the energy metabolism pathway, such as TGs, DGs, FAs, and PCs. Because previous studies have associated an elevation in TGs, DGs, FAs, and PCs with WAT browning, the observed changes in these lipid substances may also be indicative of capsaicin-induced WAT browning.

## 5. Conclusions

Spatial metabolomics represents a cutting-edge approach that integrates the spatial context of metabolite distribution within tissues. This technique stands out for its ability to provide a comprehensive and spatially resolved metabolic profile. Particularly, considering the unique anatomical structure of interscapular BAT, spatial metabolomics could be used to map the distribution of metabolites across the different regions in the interscapular BAT and its adjacent WAT. Furthermore, this could also bring a giant advantage when investigating the influence of dietary intervention on adipose tissue, which has been confirmed with strong plasticity to adapt to physiological demands and environmental changes. For instance, WAT can undergo “browning” under certain conditions, where it starts to resemble BAT in terms of its metabolic activity and mitochondrial content. Indeed, the obtained results in the current study also confirmed that capsaicin facilitates the process of browning in WAT, while notable changes in the metabolites within BAT were also observed. In conclusion, this study showed that spatial metabolomics could be used as a powerful tool to offer insights into the regulatory effects of dietary interventions on adipose tissues.

## Data Availability

The original contributions presented in this study are included in the article/Supplementary Materials. Further inquiries can be directed to the corresponding authors.

## Supplementary Materials

The following supporting information can be downloaded at: https://www.mdpi.com/article/10.3390/foods13233943/s1. Table S1: Nutritional composition of the mice feed; Table S2: List of differential metabolites between interscapular WAT and BAT in mice without capsaicin treatment; Table S3: List of differential metabolites between interscapular WAT and BAT in mice with capsaicin treatment; Table S4: List of differential metabolites between interscapular BAT (inner layer) in mice with and without capsaicin treatment; Table S5: List of differential metabolites between interscapular BAT (layer adjacent to WAT) in mice with and without capsaicin treatment; Table S6: List of differential metabolites between interscapular WAT in mice with and without capsaicin treatment.
