# Supplementary material for: Spatial Metabolomics Reveals the Effects of Dietary Capsaicin Intervention on Interscapular Adipose Tissue Metabolome in Mice
Source: Foods. 2024 Dec 6;13(23):3943. doi: 10.3390/foods13233943 (PMC11641047; doi:10.3390/foods13233943)
Supplement: Supplementary file 1 [file foods-13-03943-s001.zip › Supplementary Table S1.pdf]

Supplementary Table S1. Nutritional composition of the mice feed

Component	Content
carbohydrates	40.6%
Protein	21.1%
Glucose	20.0%
Fiber	7.7%
animal fat	4.5%
Ca	1.8%
Phosphorus	1.2%
V_D	1800 IU/Kg
V_A	13000 IU/Kg
V_E	140 IU/Kg
V_K	7 IU/Kg
Choline	1260 mg/Kg
K	0.4%
Mg	0.23%
Na	0.20%
Fe	133 mg/Kg
Mn	87 mg/Kg
Zn	30 mg/Kg
Cu	14 mg/Kg
Lys	1.8%
Arg	1.5%
Met+Cys	0.9%
His	0.8%
Trp	0.3%
Thr	0.6%
Iie	1.4%
Phe	1.6%
Leu	1.7%
Val	1.7%
